# Gastric Mixed Adenoma Well-Differentiated Neuroendocrine Tumor or Mixed Neuroendocrine–Non-neuroendocrine Neoplasm: A Case of Classification Dilemma

**DOI:** 10.14309/crj.0000000000001841

**Published:** 2025-10-01

**Authors:** Sameer Rao, Esli Medina-Morales, Riya Sutariya, Vraj Shah, Ritik Mahaveer Goyal, Vivian Purizaga, Bryan Medina-Morales, Maria Kapsoli, Harold Benites-Goñi

**Affiliations:** 1 Department of Medicine, Rutgers New Jersey Medical School, Newark, NJ; 2 Department of Gastroenterology and Hepatology, Rutgers New Jersey Medical School, Newark, NJ; 3 Department of Gastroenterology, Hospital Nacional Edgardo Rebagliati Martins, Lima, Peru; 4 Department of Pathology, Hospital Nacional Edgardo Rebagliati Martins, Lima, Peru; 5 Universidad San Ignacio de Loyola, Lima, Peru

**Keywords:** mixed neuroendocrine–non-neuroendocrine neoplasms, mixed adenoma with well-differentiated neuroendocrine tumors, gastric mixed tumor

## Abstract

Mixed neuroendocrine–non-neuroendocrine neoplasms (MiNENs) and mixed adenoma with well-differentiated neuroendocrine tumors (MANETs) are rare neoplasms composed of neuroendocrine (NE) and non-NE components. Accurate classification is essential, as MiNENs are typically aggressive, whereas MANETs typically follow an indolent course. We present a case of a gastric mixed tumor in a 69-year-old woman, comprising tubular adenoma, adenocarcinoma in situ, and a low-grade NE tumor. Although the tumor did not meet the current WHO criteria for MiNEN, it demonstrated unusually aggressive behavior including rapid progression and lymph node metastasis, atypical for a MANET. This highlights the need to refine current diagnostic classifications to better treat such mixed tumors, as MANETs are typically managed with polypectomy, whereas MiNENs may require extensive endoscopic or surgical intervention.

## INTRODUCTION

**Figure 1.**
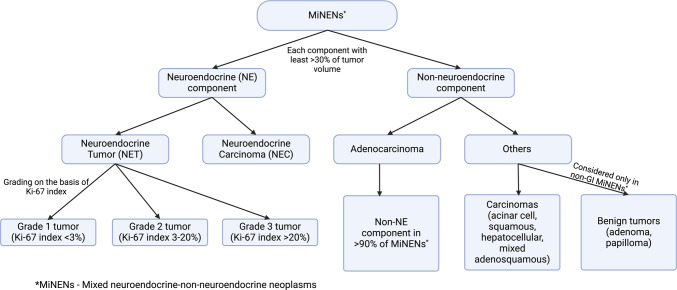
Different possible neuroendocrine (NE) and non-NE components of MiNEN based on the latest guidelines. GI, gastrointestinal; MiNENs, mixed neuroendocrine–non-neuroendocrine neoplasms.

Mixed neuroendocrine–non-neuroendocrine neoplasms (MiNENs) are tumors composed of both neuroendocrine (NE) and non-NE components, each constituting more than 30% of the tumor volume. The NE component can be a neuroendocrine tumor (NET) or a neuroendocrine carcinoma. The non-NE component may include adenocarcinomas or other carcinomas (eg, squamous cell, acinar cell, hepatocellular), as well as benign tumors (eg, adenoma, papilloma) (Figure 1).^1,2^ However, the 2019 World Health Organization (WHO) classification of digestive system tumors introduced an updated definition of lesion composition to be included within the conceptual framework of MiNEN in the gastrointestinal tract.^3^ Mixed tumors with a non-neuroendocrine (non-NE) component consisting of benign lesions, such as tubular adenomas (eg, mixed adenoma and well-differentiated NETs [MANETs]), are no longer categorized as MiNENs, because of their typically indolent behavior, requiring less aggressive treatment.^4–6^

MiNENs and MANETs are rare tumors, particularly gastric MANETs, which have thus far been reported only in case reports or small case series.^4^ We present a unique case of a 69-year-old woman with a gastric mixed tumor composed of tubular adenoma with high-grade dysplasia, gastric adenocarcinoma in situ, and a low-grade NET. To the best of our knowledge, this is the first report in the literature describing a mixed gastric tumor with this specific combination of NE and non-NE components.

## CASE REPORT

**Figure 2.**
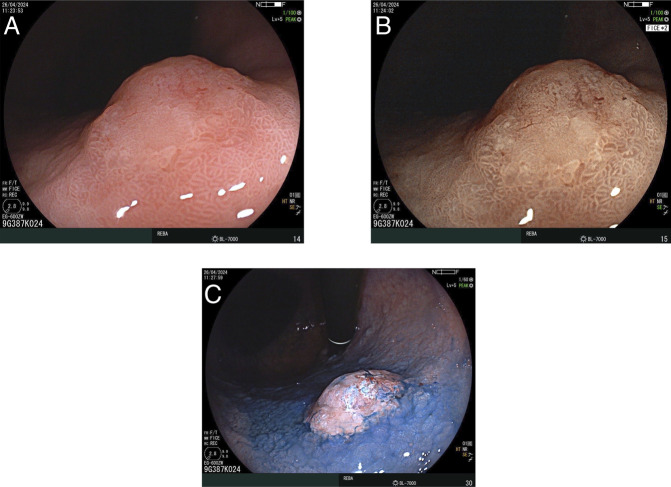
Endoscopic images of the lesion on the anterior wall of the upper body of the stomach: (A) Subepithelial lesion with an elevated center and aberrant thin vessels on the surface, (B) virtual chromoendoscopy with magnification showing no identifiable surface pattern, and (C) elevated area of the lesion highlighted using conventional chromoendoscopy with 0.3% indigo carmine.

**Figure 3.**
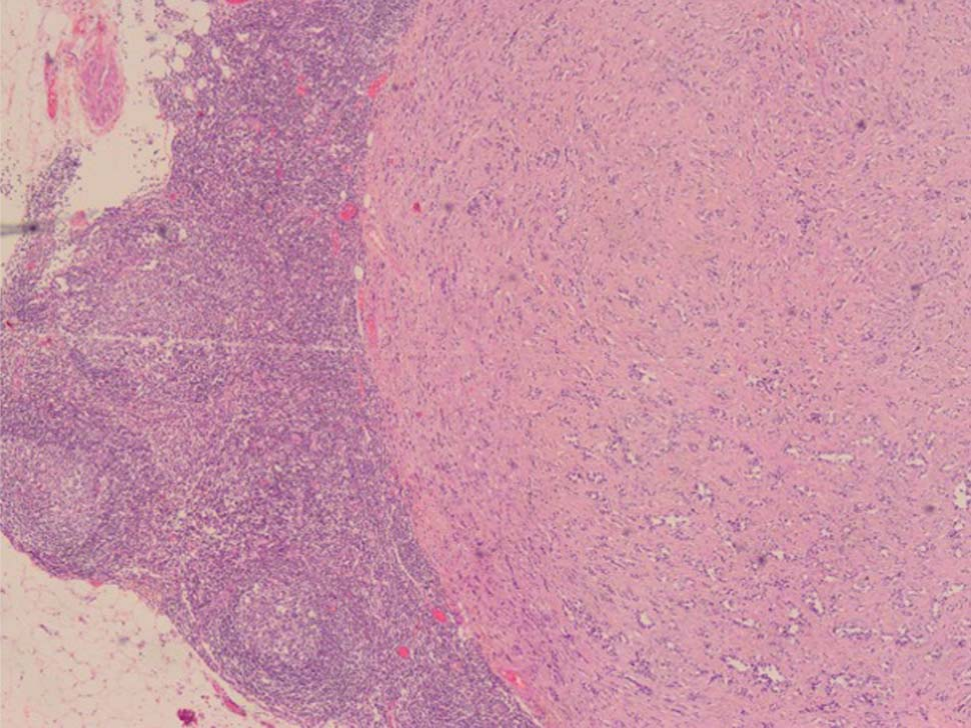
Histological images of perigastric lymph node showing involvement by a well-differentiated neuroendocrine tumor at 10× magnification.

A 69-year-old woman presented to Rebagliati Hospital in Lima, Peru, approximately 5 months after undergoing an endoscopy at an outside facility, which revealed 2 gastric lesions, with a biopsy of the larger lesion showing tubular adenoma with low-grade dysplasia. The patient reported a year-long history of bloating and diarrhea at the time of evaluation at our facility. An esophagogastroduodenoscopy performed at our center identified an 11-mm pale lesion with an elevated center and a subepithelial appearance on the anterior wall of the upper body of the stomach (Figure 2). Magnifying flexible spectral imaging color enhancement revealed a clear demarcation line, irregular microvascular and microsurface patterns, and the presence of a nonextension sign on dynamic assessment. Histological examination of the lesion showed intestinal-type gastric adenoma with areas of both low- and high-grade dysplasia. Subsequently, an endoscopic ultrasound was performed, revealing enlarged perigastric and peripancreatic lymph nodes. A biopsy of 1 of the 3 enlarged perigastric lymph nodes revealed a well-differentiated grade 1 NET (Figure 3). Computed tomography of the abdomen and pelvis showed no evidence of distant metastasis.

**Figure 4.**
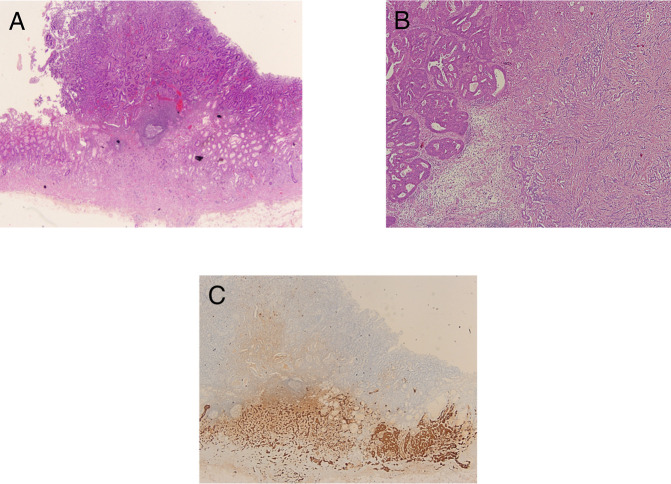
Histological examination of the polypoid lesion postgastrectomy: (A) low-magnification view (4×) showing gastric mucosa infiltrated by mixed tumor, comprising moderately differentiated tubular adenocarcinoma (upper section) and well-differentiated grade 1 neuroendocrine tumor (lower section), (B) high-magnification view (10×) displaying adenocarcinoma glands with a cribriform-like infiltration pattern (left) alongside a well-differentiated grade 1 neuroendocrine tumor (right), and (C) chromogranin immunostaining demonstrating positivity in the neuroendocrine tumor at 10× magnification.

After a multidisciplinary meeting, a decision was made to perform a laparoscopic gastrectomy with D2 lymphadenectomy. Gross examination of the resected specimen revealed a 15 × 15-mm polypoid lesion on the lesser curvature. Histological analysis confirmed a mixed tumor comprising both NE and non-NE components, with 40% tubular adenoma exhibiting high-grade dysplasia, 10% gastric adenocarcinoma in situ, and 50% NET (Figure 4). The lesion was classified as pathological stage T1bN2, with submucosal invasion measuring 1,500 μm, no evidence of ulcerative changes (pUL0), presence of lympho-vascular invasion (Ly1), absence of venous invasion (v0), and no involvement of either the horizontal margin (pHM0) or the vertical margin (pVM0). Immunostaining of the NET component was positive for chromogranin A, synaptophysin, and cluster of differentiation 56 (CD56), with a Ki-67 index of 2.27% (Figure 4).

The patient experienced no postoperative complications, tolerated oral nutrition well, and was discharged 7 days after surgery. The patient did not receive chemotherapy or radiotherapy after resection and has remained in remission during 1 year of follow-up.

## DISCUSSION

MiNENs are aggressive tumors with poor prognosis and a high recurrence rate, whereas MANENs are generally considered indolent with a more favorable outcome.^4,5,7^ Thus, distinguishing between these 2 entities is crucial. Our case does not meet the latest WHO criteria for MiNENs, which require both the NE and non-NE components to have an invasive component exceeding 30% of the tumor volume, nor does it align with MANETs as it exhibits atypical characteristics, such as the presence of adenocarcinoma in situ as one of the non-NE components.^3^ Moreover, the rapid progression from a low-grade lesion to a high-grade lesion within a few months contradicts the typically indolent nature of MANETs.^6^

Diagnostic challenges are inherent in both MiNENs and MANETs, primarily because of the need to identify both NE and non-NE components, which are often missed in endoscopic biopsies. This was also observed in our case, where the endoscopic biopsies only identified the non-NE component. A study demonstrated that presurgical biopsies detect MiNEN in only one-third of the cases, with the remaining often suggesting a pure counterpart.^7^ In addition to recognizing both NE and non-NE features on hematoxylin and eosin staining, it is crucial to use an appropriate immunohistochemical panel. Synaptophysin, chromogranin A, and CD56 are commonly used markers to identify NE differentiation and the non-NE markers include caudal-type homeobox 2 (CDX2), cytokeratin 7 (CK7), CK20, among others depending on the specific neoplasm. CDX2 is typically expressed in intestinal epithelium and serves as a marker of intestinal differentiation, whereas CK7 and CK20 expression patterns assist in distinguishing between gastric and intestinal phenotypes and can provide insight into the adenocarcinoma lineage.^2,8,9^ Emerging data suggest that mixed tumors may share a common clonal origin, with later molecular divergence driving NE and non-NE phenotypes. Although molecular profiling was not performed in our case, next-generation sequencing could clarify tumor lineage and identify drivers of aggressive behavior, informing prognosis and treatment.^6^

Recent studies have questioned the credibility of arbitrarily using 30% tumor volume for NE component as a threshold to establish the diagnosis of MiNEN.^10,11^ A retrospective study compared survival rates in 88 patients with gastric carcinomas with varying degree of NE component to 650 gastric carcinomas with no NE component. Overall, 5-year survival in cases with <10% NE component was 85.6% compared to only 53.3% survival in cases with ≥10% NE component, suggesting that ≥10% NE component threshold is associated with poor prognosis.^12^ There is a need for large-scale studies to review the diagnostic threshold to ensure that proper treatment strategies could be adopted as these tumors can be very aggressive in nature, and misclassification could be detrimental for patient care because of difference in management styles. Furthermore, the presence of adenocarcinoma in situ, a noninvasive but high-risk lesion, further complicates classification, as current WHO guidelines focus on invasive adenocarcinoma as the non-NE component.^13^

MANENs are typically managed like pure adenomas, with polypectomy as the standard therapeutic approach.^4,5^ In contrast, treatment for MiNEN is generally tailored to the most aggressive component of the tumor, which is usually neuroendocrine carcinoma. If there is a MiNEN with NET and adenocarcinoma, treatment is usually tailored toward adenocarcinoma.^14^ In nearly all cases of MiNEN, endoscopic submucosal dissection and/or surgical intervention are recommended to prevent recurrence of malignancy.^15^ In our case, because of the presence of perigastric lymph node metastasis involving the NE component, a laparoscopic gastrectomy with D2 lymphadenectomy was performed. In addition, our case report underscores the potential aggressiveness of NETs, as evidenced by lymph node metastasis occurring in a tumor with a low-grade NET component.

In our case, lymph node metastasis and rapid progression occurred despite a low Ki-67 index (<3%), highlighting a potential mismatch between histologic grade and biological aggressiveness. When such divergence is observed, clinicians may consider closer surveillance protocols, including serial endoscopy and imaging, as well as more aggressive surgical interventions, rather than limited local excision, for MANETs.

In summary, our case report highlights the heterogeneous nature of mixed neoplasms containing both NE and non-NE components. There is a critical need for multicenter prospective studies including molecular profiling to consider refining the nomenclature, address gaps in the literature on these mixed tumors, and establish standardized management strategies to improve patient outcomes.

## DISCLOSURES

Author contributions: S. Rao, E. Medina-Morales, R. Sutariya, V. Shah, B. Medina-Morales, and H. Benites-Goñi reviewed the literature and wrote the manuscript. RM Goyal, M. Kapsoli, and V. Purizaga reviewed the literature, evaluated, and provided the pathology slides and endoscopic imaging. B. Medina-Morales, M. Kapsoli, and H. Benites-Goñi participated in patient care. Rao, E. Medina-Morales, and H Benites-Goñi reviewed and edited the manuscript. H. Benitez-Goñi is the article guarantor. All authors approved the manuscript.

Financial disclosure: None to report.

Informed consent was obtained for this case report.

## ABBREVIATIONS:

CD: cluster of differentiation
CDX: caudal-type homeobox
CK: cytokeratin
MANET: mixed adenoma with well-differentiated neuroendocrine tumor
MiNEN: mixed neuroendocrine-non-neuroendocrine neoplasm
NE: neuroendocrine
NEC: neuroendocrine carcinoma
NET: neuroendocrine tumor
WHO: world health organization
